# Grounding Mental Representations in a Virtual Multi-Level Functional Framework

**DOI:** 10.5334/joc.249

**Published:** 2023-01-12

**Authors:** Pierre Bonzon

**Affiliations:** 1 University of Lausanne, Faculty of Economics, Dept of Information Systems, CH

**Keywords:** cognitive development, mental representation, representational vehicle, representational contents, associative memory, virtual machine

## Abstract

According to the associative theory of learning, reactive behaviors described by stimulus-response pairs result in the progressive wiring of a plastic brain. In contrast, flexible behaviors are supposedly driven by neurologically grounded mental states that involve computations on informational contents. These theories appear complementary, but are generally opposed to each other. The former is favored by neuro-scientists who explore the low-level biological processes supporting cognition, and the later by cognitive psychologists who look for higher-level structures. This situation can be clarified through an analysis that independently defines abstract neurological and informational functionalities, and then relate them through a virtual interface.

This framework is validated through a modeling of the first stage of Piaget’s cognitive development theory, whose reported end experiments demonstrate the emergence of mental representations of object displacements. The neural correlates grounding this emergence are given in the isomorphic format of an *associative memory*. As a child’s exploration of the world progresses, his mental models will eventually include representations of space, time and causality. Only then epistemological concepts, such as *beliefs*, will give rise to higher level mental representations in a possibly richer propositional format. This raises the question of which additional neurological functionalities, if any, would be required in order to include these extensions into a comprehensive grounded model. We relay previously expressed views, which in summary hypothesize that the *ability to learn* has evolved from *associative reflexes and memories*, to suggest that the functionality of *associative memories* could well provide the sufficient means for grounding cognitive capacities.

## 1. Introduction

Following the advent of cognitive science, psychology has come to be described as the study of mental representations, how they are computed, and how they affect behavior (Gallistel, 2001). According to widely accepted views, mental representations are internal brain states and mechanisms that drive flexible behaviors i.e., behaviors that cannot be explained in terms of stimulus-responses only. There is still no consensus however on how mental representations are grounded in the brain, and especially about the nature of the computation they involve (Gallistel, 2017; Smortchkova et al., 2020).

Recent significant contributions in this domain go back to Haugeland (1991), who did characterize a representational system as encompassing substitutes for environmental signals. In the same vein, Vosgerau (2010) calls for a “functionalistic core” in which representations are substituted as arguments in templates that are stored in memory. Bechtel (2016) focuses on the neural processes in which mental representations can be grounded and describes them as *vehicles* carrying *contents* e.g., spatial information that can be processed. Ramsey (2016) emphasizes their *functional role* and specific *content*, which are related to each other by virtue of causal links. To the contrary, Egan (2020) advocates a “deflationary” account of mental representation, which in summary denies them any intrinsic or naturalistically determinate content, their representational capacity being then defined by a function projecting from the level of neural structures to that of behaviors. Consensually, Orlandi (2020) presents a set of necessary and sufficient conditions for internal states or structures to qualify as mental representations, their role being then essentially to serve as *isomorphic* stand-ins that produce stimulus-free behavior. To wrap things together, Newen & Vosgerau (2020) first develop a “functionalist framework” in which behaviors are defined as mappings from stimulus/state to state/response pairs; mental representations are then viewed as substitutes for arguments, their coarse grained contents being determined by their functional role, and their vehicle standing as neural correlates; in order to reflect different cognitive explanatory levels, they finally introduce a mental representation’s third dimension i.e., its “fine-grained structure”, which could be either in a *correlational, isomorphic*, or *propositional format*.

In summary, mental representation can be characterized by distinguishing their vehicles from their contents. Whereas it is commonly agreed that these vehicles, which carry some informational contents, are constituted by neural correlates, the true nature of these contents themselves is still a subject of controversy (Egan 2014; Ramsey 2020). So-called “deflationary” accounts of mental representations tend to consider them as void of any intrinsic or naturalistically determinate content. Without grounding them, theories of mental representation postulate the existence of causal structures and processes, which the brain actually progressively constructs in its interaction with the world. The shortcomings of this approach might thus well be to put the cart before the horse. What is needed in contrast is a analysis that allows first for *independently* abstracting low-level functionalities of the brain, on one hand, and higher-level informational links between these functionalities and behaviors, on the other, and then for *relating* them through a meso-scale level virtual interface.

## 2. Goals and results

This work has the following three goals:

1. clarify the status of mental representations through a model relating their neurological and informational functionalities;
2. validate this model through an effective simulation of reported experiments demonstrating the early emergence of mental representations;
3. address the question of which low-level brain functionalities would be required for grounding this model into biological structures and processes.

Concerning goal 1), we shall argue that the controversy about deflationary accounts of mental representations (Egan 2020; Hutto & Myin 2020; Ramsey 2020) can be clarified by providing a multi-level analysis of foundational concepts. Firstly, the vehicles of mental representations have a *physical* support constituted by neural correlates that supposedly carry some informational contents. Secondly, these contents have a *functional* role, which is to serve as substitutes for arguments in the processes that support cognition. Finally, these cognitive processes themselves are meant to support flexible behaviors. To formalize this procedural hierarchy, the relation between the brain substrate and cognitive processes on one hand, and between cognitive processes and behaviors, on the other, will be abstracted using tools from the domain of computer science, namely *concurrent communicating systems* and *virtual machines*. Concurrent communicating systems model the interaction of objects obeying various communication protocols, and thus reflect a high level view of a neural network i.e., of the brain substrate. The concept of a *virtual machine* interpreting a compiled code that differs from a processor’s native code constitutes the key mechanism that allows for interfacing high level abstract objects i.e., software, with their low level physical support i.e., hardware. Following classical results of computer science, symbolic expressions that have been compiled and then interpreted by a virtual machine get their operational semantics from the transitions they induce on the state of this machine. In the context of a multi-level model of brain structures and processes, this means that low levels physiological details can be ignored, and grounded models of cognition can be formulated by relating input and output (i.e., perception and behavior) at a *symbolic level*. Furthermore, as a program can modify itself as well as the virtual machine, the whole system is evolutive by virtue of its very nature. As a result, and similarly to software made of successive layers of programs that are dynamically interpreted and ultimately executed on hardware, this formalism allows for modeling behaviors driven by successive layers of cognitive processes that are ultimately grounded in the brain.

To validate this proposition and satisfy goal 2), we shall draw on an implementation of Newen & Vosgerau (2020) overall functionalist framework. Behaviors, which in this framework are defined as mappings from one augmented state of mind to another, explicitly interacts with the environment, and thus allow for dynamically constructing mental representations on the fly. A simulation of the initial *sensory-motor stage* of Piaget (1937) cognitive development theory is proposed as an illustrative case study. It must be stressed from the onset that this simulation is not meant to be a comprehensive model of the Piagetian theory. The storage and retrieval mechanisms involved in the process demonstrate how, at this stage of cognitive development, neural correlates of representational vehicles implement the functionalities of an *associative memory*. This supports the hypothesis that, still at this initial stage, “*mental models are structures partially isomorphic to what they represent and that they contain exclusively perceptual relations*” (Vosgerau 2006). As a child’s exploration of the world progresses, his mental models will eventually include representations of space, time and causality. Only then epistemological concepts, such as *beliefs*, will give rise to higher level mental representations in a possibly richer format. This directly leads to the question raised in goal 3) i.e., which additional low level brain functionality, if any, would be needed in order to accommodate these extensions in a simulation, and thus achieve a comprehensive model of cognitive development. Our answer to this question relays prospective views previously suggested by some authors, and which essentially amount to hypothesize that evolution might have been satisfied with the first and simple solution it did encounter for grounding emergent mental representations.

## 3. Defining mental representations in a functionalist framework

In order to situate our solution to goal 1), we shall first provide an historical survey of past developments that occurred in related research domains. This will then lead us to formulate in turn two questions related to fundamental methodological issues.

### 3.1 Historical background

As a result of its cognitive revolution, psychology has moved away from behaviorist explanations towards the study of the mind as a computational device. Behaviorism was founded on the idea that the minds of humans and non-human animals alike have to be considered as black boxes i.e., as systems for which one does not postulate anything about the processes that control them. Behaviors were then defined solely by the input-output relations that associate perceptions and actions, with observed behaviors giving rise to behavioral rules i.e., synthetic ways of expressing specific input/output relations. Behaviorist studies culminated in the throughout exploration of *operant conditioning*. In contrast, according to cognitivist views, behaviors are driven by *mental states*, which are themselves defined by brain internal structures and processes. At first sight, these two approaches seem complementary; however, behaviorism was eventually felt incompatible with the concept of a mental state e.g., by arguing that different sets of successive mental states could produce the same input-output relation i.e., would be *equivalent* without being equal.

In order to reconcile these two approaches, one could try and ground them both in a common abstract biological substrate. This would then allow for answering the question of which neural structure could possibly drive an observed behavior, or other words, which neural structure could possibly be associated with a given behavioral rule. Associate learning has been linked to the neural processes of long term potentiation/depreciation (Bliss & Lomo 1973; Markram et al. 1997; Brette et al. 2007). This provides cues of what cognitive states could be made of, and constitutes a possible starting point for theoretically grounding cognitive computations. It raises in turn two related questions i.e.:

1. what is the right level of analysis for grounding cognitive computations;
2. what might be a suitable formalism for interfacing cognition and behaviors?

### 3.2 What is the right level of analysis for grounding cognitive computations?

As argued by Marr (1982) in his “tri-level” hypothesis, the “*what*” and “*how*” of cognitive science can be described in *computational, algorithmic* and *implementation* terms. An exploration of this hypothesis and of its relation to Cognitive Science has been intensively studied (see e.g., Cooper & Peebles 2015; Love 2015).

The early computational models of cognition (see e.g., Newell et al. 1989; Anderson et al. 2004) were symbolic information processors originating from the research in Artificial Intelligence. Based on hypotheses about how humans are supposed to behave, these *top down* approaches did allow for performing simulations regardless (at least at an earlier stage) of any grounding issues i.e., they did not address the problem of binding behavior to the brain. In parallel, another field of research focused on brain structures and processes. Numerous computational platforms aiming at simulating networks of neurons and ranging from artificial neural networks (see e.g., Rumelhart & McLelland 1986; Hinton et al. 2006) based on a threshold logic (McCulloch & Pitts 1943) to analytical neuron simulations (see e.g., Hines and Carnevale 1997; Markram et al. 2015) based on differential equations (Hodgkin & Huxley 1952) to simulate currents have been proposed. These constitute *bottom up* approaches detailing the ground level constituents of the brain (i.e., the neuronal cells and their connections), and as such are not explicitly related to the cognitive tasks they support.

So-called “neurally plausible cognitive architectures” later tried to associate cognitive models with some form of brain modeling. Designated as hybrid *symbolic connectionist models* (see e.g., Shastri & Ajjanagadde 1993; Hummel & Holyoak 2005; Doumas et al. 2008), they constitute an attempt to combine the conceptual simplicity and the computing power of artificial neural networks with the expressive capabilities of the models of Artificial Intelligence, and allow in particular for dealing with symbols. According however to a notable attempt using an analytic neuron model (Jilk et al. 2008), “the incommensurable categories at the various levels of description will remain necessary to explain the full range of phenomena”, meaning that it is illusory to try and reduce the complexity of the brain to a single level of description. As pointed by many authors (Carandini 2012; Love 2015; Forstmann & Wagenmakers 2015; Cooper & Peebles 2015), formal models are needed to provide links between the brain substrate and cognitive processes.

The growing availability of neural data from brain imaging techniques has given rise to a new interdisciplinary field i.e., *model-based cognitive neuroscience* (Mulder et al. 2014; Forstmann & Wagenmakers 2015; Palmieri et al. 2017). Very broadly, these approaches rely on statistical methods to relate data to patterns of neural activity. As an example, Seth et al. (2004) study causal interactions in neural populations by applying a combination of time series analysis and graph theory; Borst & Anderson (2017) use ACT-R with fMRI data to predict the neural correlates of constrained model processes. In order to characterize different such approaches Turner et al. (2017) consider two data domains, namely neural data denoted by ***N*** and behavioral data, denoted by ***B***, and distinguish three ways these two domains can interact i.e.,

1. using the neural data to constrain a behavioral model
2. using the behavioral model to predict neural data
3. modeling both neural and behavioral data simultaneously.

All these approaches rely on statistical methods to relate data to patterns of neural activity. Whereas the first two cases use unidirectional statistical influence, the third one relies on a bidirectional link between measures of different modes to formalize a connection between ***N*** and ***B*** through a cognitive model. As a further characterization of this third case, Turner et al. (2017) distinguish two sub cases, namely the *joint modeling* and the *integrative* approaches. In order to relate the model parameters *δ* and *θ* that respectively predicts ***N*** and ***B***, the joint modeling approach consider higher level parameters *Ω* linking *δ* and *θ* in a hierarchical Bayesian structure connecting the neural and behavioral levels (Turner 2015). In contrast, the integrative approach relies on a single set of parameters e.g., the sequence of module activations in the ACT-R framework that assumes a predefined set of modules.

At first sight, the joint modeling approach allows for a projection into Marr’s three-level analysis concepts i.e., the computational and algorithmic levels being constituted respectively by a Bayesian probabilistic model and an inference rule. The mere existence of a corresponding neuronal implementation is however highly hypothetical. The complexity of the corresponding neural phenomena has so far prevented the definition of models directly relating single neuron dynamics to global brain states (see e.g., Goldman et al. 2019). As an example, whereas neurological measurements (Tomov et al 2018) have validated a computational Bayesian model positing a dedicated neural mechanism for causal learning (Gershman 2017), nothing is known about the corresponding basic neuronal processes.

In any case, as argued thoroughly by Frégnac (2017), “big data is not knowledge”. The causal link between sub-cellular/cellular mechanisms and behavior should be achieved through successive levels of analysis, as exemplified by Marr’s hypothesis. This means that mappings need to be expressed in *algorithmic* terms and not just in a correlative way. In order to take into account intermediate levels of circuit integration, canonical operations should be defined as *invariant* computations. As discussed in (Frégnac & Bathellier, 2015), top-down approaches via analytical and/or abstract mathematical tools such as Bayesian inference rules (see e.g., Ma and Pouget 2008), and for that matter we may add the bottom-up approaches of the classical theories based on artificial neural networks (Kohonen 1982; Hopfield 1982) as well as methods related to dynamical systems theory and neural fields (Thelen & Smith 1994; van Gelder 1998), are well suited for describing computations in Marr’s sense, but *“fail to identify algorithms and underlying circuits”*. What is then needed, they conclude, is a ‘‘*middle-out*’’ approach that can identify plausible structures and processes linking biology and cognition across successive levels of integration distinguishing *micro-scale* and *meso-scale* functions. Analogous conclusions can be found in the insightful review of van der Velde and de Kamps (2015), who argue that cognitive processes are executed in connection structures that link sensory circuits (i.e., perception) with motor (i.e., action). What is needed, they add, is “a mechanism that shows how the information (*synchrony* of activation in this case) can be used by the brain”.

In conclusion, there does not seem to be a single level for grounding cognition, and the problem is rather to design an *interface* between different levels of study.

### 3.3 What might be a suitable formalism for interfacing cognition and behaviors?

Looking at the brain as a computing device linking neural dynamics to actions has led to the emergence of quite a few related research domains. Whereas *computational neuroscience* addresses low level neural mechanisms that give rise to higher level processes representing computations, *cognitive neuroscience* attempts to relate brain and behavior by linking latent cognitive processes to the neural mechanisms that generate them (Frank and Badre 2015). These two disciplines, when taken together, form the *computational cognitive neuroscience* (or CCN) paradigm (O’Reilly and Munakata 2000; Ashby and Helie 2011; Kriegeskorte & Douglas 2018), in which artificial neural network models and methods serve *both* to specify and to concretize theories (Herd et al. 2013). A cognitive model however doesn’t have to represent its underlying neuronal processes itself, as the present approach to CCN does, but could rather adds an intermediate layer between the neuronal and behavioral layer (Mulder et al. 2014; Frank 2015), using formal models to connect findings from neuroscience to the cognitive processes at hand (Forstmann and Wagenmakers 2015).

The interface between these various layers could be described using computer science methods that allow for a delineation and implementation of successive levels of complexity. Present day computer software methodology follows a two levels approach: application source programs written in a high level language (e.g., Java) get interpreted by an intermediate program constituting a *virtual machine*, which itself gets executed as an object program written in a processor native code. This mechanism constitutes the key mechanism that allows for interfacing two independent objects i.e., *software* and *hardware*. In the context of a multi-level model of brain structures and processes, this means that low levels physiological details could be ignored, and grounded models of cognition be formulated by relating input and output (i.e., perception and behavior) at a *symbolic level*.

This is the approach that will be illustrated here. As computer applications can be first programmed, then compiled and finally interpreted by a virtual machine running as a native program, cognitive processes will be similarly encoded, compiled and then interpreted by virtual neurological microcircuits representing a brain’s innate processes. As a consequence, there will be no reference to any specific neural network model. In contrast to the usual approach of creating neural models of interactive brain areas by quantitatively fitting data (i.e., where latent estimated parameters are being correlated with neural measures), the goal here is to construct a model of how behaviors can be interfaced with neural dynamics in order to try and discover the learning processes involved in the emergence of cognition.

## 4. A virtual neurological formalism

In the formalism presented in (Bonzon 2017; Bonzon 2019), brain processes representing synaptic plasticity are abstracted through asynchronous communication protocols and implemented as virtual *microcircuits*. The basic units of these micro-circuits are constituted by *threads*, which correspond either to a single or to a cluster of connected neurons. Contrary to traditional neuron models in which incoming signals are summed in some integrated value, thread inputs can be processed individually, thus allowing for threads to maintain parallel asynchronous communications reflecting a massively asynchronous organ (Zeki 2015). Threads can be grouped into disjoint sets, or *streams* to model neural assemblies (Gerstner & Kisler 2002; Huyck & Passmore 2013) and discrete *weights* (e.g., integer numbers) can be attached to pairs of threads that communicate within the same stream. Meso-scale *virtual circuits* linking perceptions and actions are built out of microcircuits. Circuits can be compiled into *virtual code implications* that are used just in time to deduce instructions to be finally interpreted by the *virtual machine* performing contextual deductions.

### 4.1 Basic concepts

**Figure 1:**
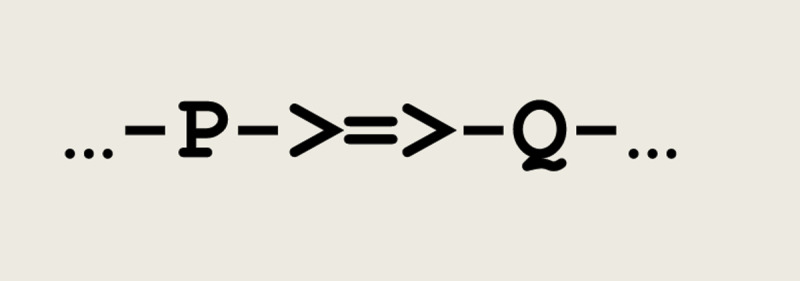
Microcircuit implementing a synaptic transmission.

To introduce this formalism, let us consider a simple case of synaptic transmission between any two threads **P** and **Q**. This can be represented by the microcircuit given in Figure 1, where the symbol **->=>-** represents a synapse.

**Figure 2:**
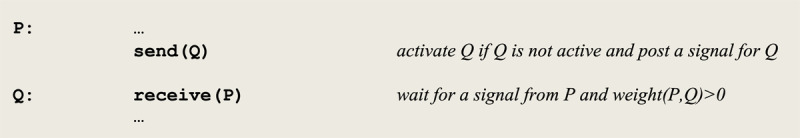
Virtual machine instructions for an asynchronous communication.

When compiled, this microcircuit will give rise to the execution of virtual machine instructions implementing the communication protocol given in Figure 2.

This protocol corresponds to an *asynchronous* communication, where a predefined weight between the sender **P** and the receiver **Q** that can be either incremented or decremented. On one side, **t**hread **P** fires thread **Q** and sends it a signal. On the other side, **Q** waits for the reception of a signal from **P** and proceeds only if the weight between **P** and **Q** stands above a given threshold. The overall process allows for passing data through variable parameters.

**Figure 3:**
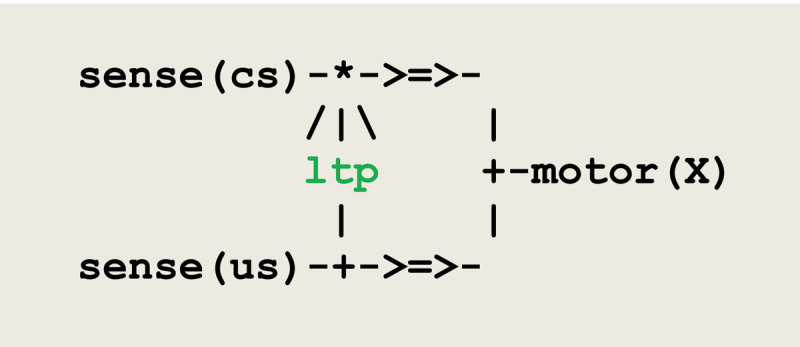
A circuit implementing classical conditioning.

As a first example, let us consider the classical conditioning of *aplysia californica* (Kandel & Tauc, 1965). In this experiment, a tactile conditioned stimulus **cs** elicits a weak defensive reflex, and an electrical unconditioned stimulus **us** produces a massive withdrawal reflex. After a few pairings of **cs** and **us** with **cs** slightly preceding **us**, **cs** alone triggers a significantly enhanced withdrawal reflex. The corresponding circuit, adapted from Carew et al. (1981), is represented in Figure 3. In this circuit, the symbol **/|\** represents the modulation of a synaptic transmission, the sign * indicates the conjunction of converging signals, and the sign + either the splitting of a diverging signal, or a choice between converging signals. The variable parameter **X** in thread **motor(X)** gets instantiated into either **cs** or **us**.

Thread **ltp** (standing for *long term potentiation*) acts as an interneuron reinforcing the pathway between **sense(cs)** and **motor(X)**.

Classical conditioning follows from the application of hebbian learning i.e., “neurons that fire together wire together”. Though it is admitted today that classical conditioning in *aplysia* is mediated by multiple neuronal mechanisms including a postsynaptic retroaction on a presynaptic site (Glanzman et al, 1995; Antonov et al, 2003), the important issue is that this activity depends on the temporal pairing of the conditioned and unconditioned stimuli, which leads to implement the thread **ltp** as a *detector of coincidence*.

### 4.2 A mechanism for simulating long term potentiation

**Figure 4:**
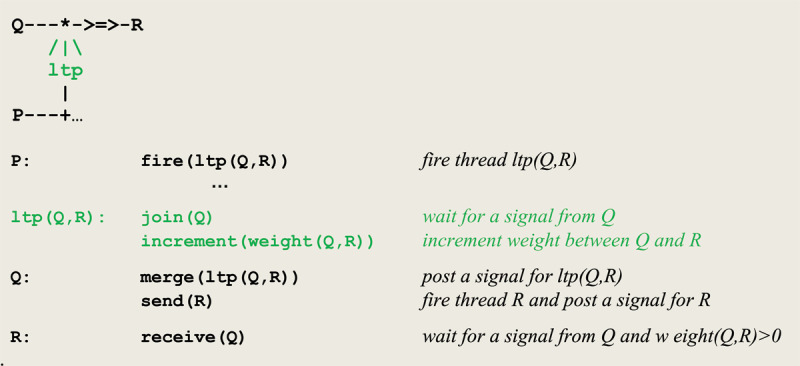
Micro-circuit and virtual machine instructions for *ltp*.

The microcircuit abstracting the mechanism of long term potentiation is given in Figure 4. As a further theoretical abstraction, our formalism allows to distinguish between a hypothetical *weak* and a *strong* synaptic plasticity reflecting brain maturation (Bolton et al 2017). Whereas *weak* plasticity binds a given sensory input with a unique reaction, a *strong* synaptic plasticity allows for associating the same input with successive different reactions.

N.B. In our implementation, this abstraction follows from the underlying logical programming framework that distinguishes between the so-called *anonymous* variables, denoted by the character “_”, and *named* variables, such as **X**, **Y**, **Z**. Whereas anonymous variables, once they get bound, do not retain their value and thus cannot create successive associations, named variables do.

In order to detect the coincidence of **P** and **Q**, **P** fires an **ltp** thread that calls on **join** to wait for a signal from **Q**. In parallel, **Q** calls on **merge** to post a signal for **ltp** and then executes a **send(R)** command to establish a link with **R**. After its synchronization with **Q**, **ltp** increments the weight between **Q** and **R**.

As a further example of long term potentiation implementing a simple form of *operant conditioning*, implying in this case learning the choice of a preferred action through a possible reinforcement, let us first introduce:

- a **watch(I)** thread that drives a learning process, where **I** is a sensory input
- a **spot(I)** thread discriminating perceptions through an *excite* or an *inhibit* stimulus
- two effector threads **accept(I)** and **reject(I)** defining output responses.

**Figure 5:**
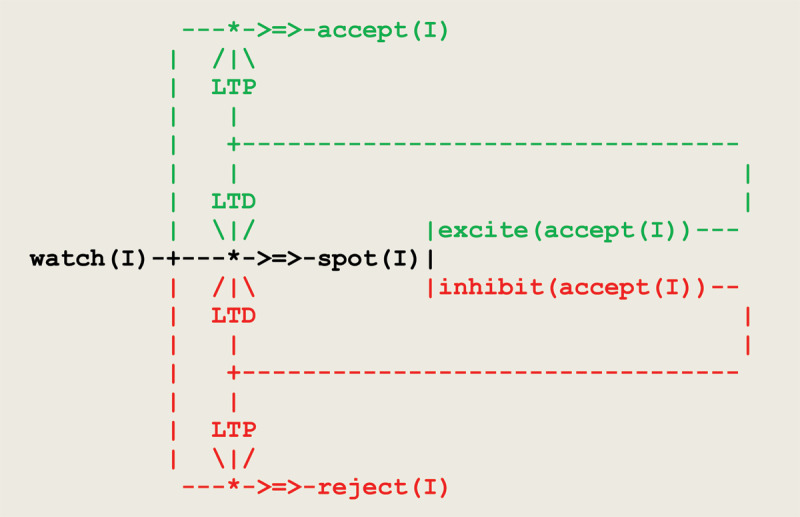
Virtual circuit implementing a simple case of operant conditioning.

Let us then consider the virtual circuit in Figure 5:

- at the start, the pathway from **watch** to **spot** is open, and pathways to **accept** and **reject** are closed;
- thread **spot** discriminates inputs through positive and negative stimuli, and thus allows for diverging paths;
- **LTP** threads open the path to either **accept** or **reject**, and **LTD** threads close the path to **spot**.

This circuit matches a fundamental principle in circuit neuroscience according to which, as a result of synaptic plasticity (expressed here through **LTP/LTD** threads), *inhibition* in neuronal networks during baseline conditions allows in turn for *disinhibition* and constitutes a key mechanism for learning (Letzkus et al 2015; Zagha et al 2015). As a result, this circuit learns a deterministic behavior driven by two neural populations competing for a response.

### 4.3 An extended associative mechanism for memory engrams

According to experimental results (Liu et al. 2012; Ryan et al., 2015; Tonegawa 2015), memory engrams involve two different circuits and mechanisms i.e., the retention of specific patterns of connectivity between engram cells required for the storage of information, on one hand, and the synaptic strengthening needed for its consolidation and retrieval, on the other.

**Figure 6:**
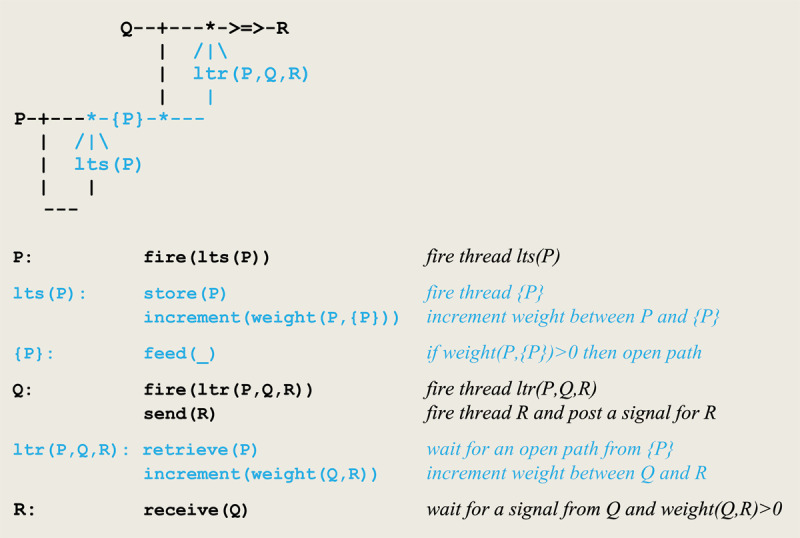
Microcircuit and virtual machine instructions for a long term associative memory.

In order to allow for two threads **P** and **Q** attached to separate streams and active at different times to be associated in order to trigger a recall thread **R**, a new communication protocol involves two complementary *long term storage/retrieval* **(lts/ltr)** threads. This new protocol, which leads to the building of a storage trace depicted by **-{P}-** and its later retrieval, is given in Figure 6. As a distinctive difference from an **ltp(Q,R)** thread (which gets fired by **P** and waits for a signal from **Q** in order to relate **Q** and **R**), an **ltr(P,Q,R)** thread is fired by **Q** and waits for a path from **{P}** in order to relate **Q** and **R**, thus defining the basic mechanism of an *associative memory*.

## 5. A virtual machine formalism

The concept of a virtual machine that we shall use allows for emulating the execution of a program given in a *symbolic* language *S* on a system having its own *logical* language *L*. On the cognitive side, *virtual circuits*, which somehow correspond to cognitive software written in language *S*, are compiled into virtual code implications of language *L*. On the neural side, these implications are used in turn to deduce just in time instructions that get interpreted by the virtual machine i.e., this virtual machine actually performs *contextual deductions* (Bonzon et al., 2000). In addition, languages *I* and *O* define respectively *input/output* sentences captured by *sensors* and delivered to *effectors*. By developing how models are actually constructed, we get the following functional signatures:

***compile: S***→ ***L***

***load: L***× ***L***→ ***L***

***compile and load: S*** × ***(S***→ ***L)*** × ***L***→ ***L***

***compile load and run: I*** × ***(S***× ***(S*** → ***L)*** × ***L)*** → ***L***× ***O***

Running a compiled model on a virtual machine then defines the reduced signature:

***run: I*** × ***L*** → ***L***× ***O***

In the realm of behaviorism, reactive behaviors are described by mappings from inputs (stimuli) to outputs (responses). In the realm of cognitivism, these mappings are extended in order to account for flexible behaviors. According to Newen & Vosgerau (2020), the corresponding functions take two arguments i.e., a stimulus/internal state pair, and produce two values i.e., a resulting internal state/response pair. The reduced signature of the *run* mapping as defined above corresponds to the one called for by Newen & Vosgerau.

Let *Model ϵ L, Input ϵ I* and *Output ϵ O* designate respectively an internal state, a stimulus and a response. In its interaction with the outside world, this machine does function as a non-deterministic learning automaton that repeats a *sense-react* cycle of embodied cognition (Brooks 1991). At the top level, the virtual machine is defined by a *run* procedure that consists of a *loop* whose cycle comprises a *sense* procedure followed by a *react* procedure:

*run(Model)*

    ***loop*** *sense(Model)*

                     *react (Model)*

At the next level, the *sense* procedure monitors stimuli directed to sensor threads. After capturing an *Input* interrupt, it updates *Model* through a transition function *input*:

*sense(Model)*

    ***if*** *interrupt(Input)*

    ***then*** *input(Model, Input)*

The *react* procedure consists of a loop using implications in *Model* to first deduce a response under the form of a virtual machine instruction *Output*, and then updates *Model* through a transition function *output* corresponding to the execution of this virtual machine instruction:

*react(Model)*

    ***for each*** *(Instruction)*

    ***such that*** *ist(Model, Output)*

    ***do*** *output(Model, Output)*

The *ist* predicate i.e., “is true”, implements contextual deduction. As there is no specified final state, whichever state the machine is in at any given time is acceptable and represents the simulated subject’s current state of mind.

## 5. Case study: Piaget revisited

The purpose of this case study is to demonstrate how mental representations arising in the early course of a child’s cognitive development can be simulated in a computational framework. These simulations, which reproduce experiments from Piaget (1937), are not intended to be a comprehensive model of the corresponding psychological theory, but merely to explore the possible neurological structures and processes that support this development.

### 5.1 Background and scope

According to Piaget’s theory, cognitive development starts with a sensory-motor stage, which itself extends over 6 substages. At the beginning, infant behaviors are driven by elementary action schemas. Substage 4 marked with of object permanence, or *objectification* i.e., the emergence of objects as autonomous and permanent entities. In Piaget’s terminology, *assimilation* (i.e., the insertion of new perceptions into existing schemas) is followed by *accommodation* (i.e., the modification and/or extension of existing schemas). His postulate then reads as follows: “*The criterion of this objectification, hence of this rupture in continuity between things perceived and the elementary sensory-motor schemata, is the advent of the behavior patterns related to absent pictures: search for the vanished object, belief in its permanence, evocation, etc*.” (Piaget 1937, p. 5).

This theory has been repeatedly challenged (Bower 1967; Bower & Wishart 1972), especially after the evidence provided by new type of experiments did suggest that infants demonstrate an understanding of object permanence at an earlier age (Baillargeon et al 1985; Baillargeon 1987). This evidence however, which relied on a violation of the *expectation paradigm*, has been later questioned by a theoretical account of this paradigm that does not invoke object concepts (Schöner & Thelen 2006). It was consequently proposed that infant’s failure to achieve Piaget’s search tasks should not be attributed to an inadequate level of cognitive development, but rather to an inherent difficulty in coordinating perceptive and motor actions.

This controversy raises the issues of how schemas are acquired, coordinated, and eventually give rise to mental representations. Cognitive neuroscience, which aims at linking cognition to brain processes, hasn’t succeeded yet in grounding such schemas into actual neural circuits. Our formalism of symbolic neural dynamics is used here to model them as virtual circuits linking sensory inputs with perceptual responses. These circuits will be then interpreted in turn by a virtual machine thus simulating successive the substages of the sensory-motor stage.

### 5.2 Modeling reflexes (sensory-motor substages 1–2)

The observations related to the first and second sensory-motor substages have been reported in (Piaget 1936). These essentially consist in describing reflex behaviors that are driven by visual attention and culminate in coordinated prehension: the grasping of objects becomes “*systematic when the object and the hand are perceived in the same visual field”*. In other terms, following a reciprocal assimilation, “*all that is to be seen is also to be grasped and all that is to be grasped is also to be seen”*.

The work of Wible et al. (2020) offers a model of visual attention that simulates behavioral and neural correlates as the product of attractor states in a dynamical system. The model proposed here is based on symbolic neural dynamics and distinguishes two intervened steps:

- first *sensation* i.e., the capture of visual data through *sensors*
- then *perception* i.e., the interpretation of these data through virtual circuits linked to *effectors*.

Visual input data consist in an object *image* and its *position* in space. The capture of an object’s image results from multilayered neural processes taking place in the eye’s retina. As it has been demonstrated in rodent animals (O’Keefe & Dostrosky 1971; Moser et al., 2008; Moser & Moser 2008), the capture of position data is achieved via multiple receptive fields i.e., *place, head direction, grid* and *border* cells. The perception associating these two data results from yet mostly unknown higher level circuits and mechanisms (Lewis et al 2019; Bicanski & Burgess 2019; Anselmi et al 2020). Our model relies on two simplifying hypotheses:

- space will be restricted and defined as a *one dimensional* axis, with visual sensory inputs defined as **P(X)**, where **P** and **X** stand respectively for the stored image of an object and its position on the space axis, which together constitute a *numerical iden*tity i.e., a prerequisite for object permanence (Moore & Meltzoff 2004)
- neural assemblies processing these inputs will be represented by threads activated through short term *potentiation*.

**Figure 7:**
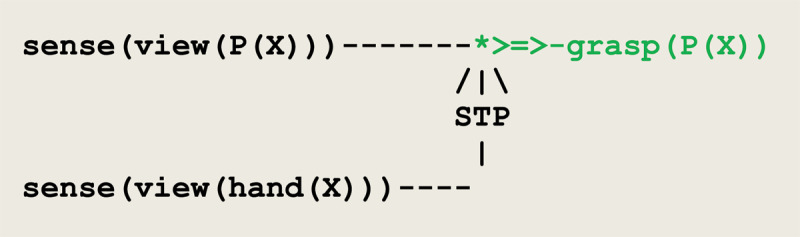
Virtual circuit implementing the grasping of an object.

On this basis, a grasping reflex can be driven by the virtual circuit given in Figure 1. In this circuit, two sensors **sense(view(P(X)))** and **sense(view(hand(X)))** converge to signal that an object **P** and a hand are perceived in the same visual field **X**. As a result, the effector **grasp(P(X))** gets activated through a short term potentiation. In conjunction with this visual drive, a grasping reflex involves other multi-modal perceptions e.g., for controlling motor actions (see e.g., Thelen et al 2001; Bonzon 2020). In the developments that follow, it is assumed that the firing of the circuit in Figure 7 will be followed by the subject’s required coordinated motor actions.

A short term potentiation **STP** opens the path from **sense(view(P(X)))** to **grasp**.

### 5.3 Modeling visual object tracking (sensory-motor substage 3)

Among others explorations, infant early experiences with the world follow from their visual attention being caught by moving objects. The tracking of moving objects results from eye saccades i.e., target-driven reflex movements. These are driven by expected upcoming data relying on pattern recognition from preceding inputs (Bicanski & Burgess 2019). As noted by Piaget (1937, p.13), “*Visual accommodation to rapid movements makes possible the anticipation of future positions of the object*”. In the case of a single object, this anticipation relies on the *focus* of attention (i.e., the position where the object is expected to next hit the eyes). When the actual sensation does not meet the expectation (i.e., if another object actually hit the eyes), visual attention gets suspended, and a default action is taken. This represents a first example of *assimilation* (i.e., in this case of the inputs produced by the moving object), followed by an *accommodation* (in this case by giving up tracking and looking instead at the occluding screen).

As an example, let us consider a simple simulation scenario that reproduces a characteristic behavior that can be observed in the third sensory-motor substage, defined as follows:

- a toy is seen moving (e.g., carried or rolling) along a one dimensional axis
- if it stands still (e.g., is dropped or stops) in the sight of the observer, he grasps it
- if it disappears behind/under a screen/object, the observer looks at the occluding item.

**Figure 8:**
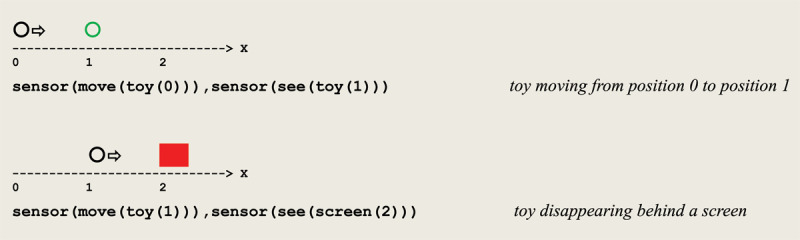
Successive eye saccades tracking a moving object.

Two successive eye saccades are sketched in Figure 8 together with their sensory input vectors.

**Figure 9:**
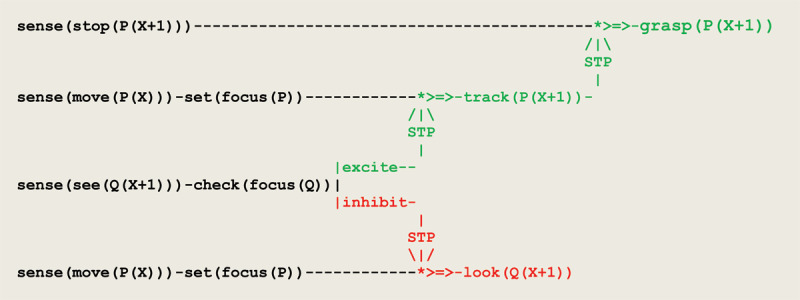
Virtual circuit implementing the visual tracking of a moving object.

As a result of discriminating inputs to eye saccades, a forward propagation of *excite/inhibit* stimuli leads to the virtual circuit implementing visual tracking given in Figure 9.

- two parallel sensor threads first process the input **move(P(X))** and set the current focus of attention to **P**;
- these two threads then wait for a sensor thread to process the input **see(Q(X+1))**;
- current focus of attention leads to apply a short term potentiation to one of two threads;
- signal that the toy stands in the observer’s visual field leads to a **grasp(P(X+1))**.

**Figure 10:**
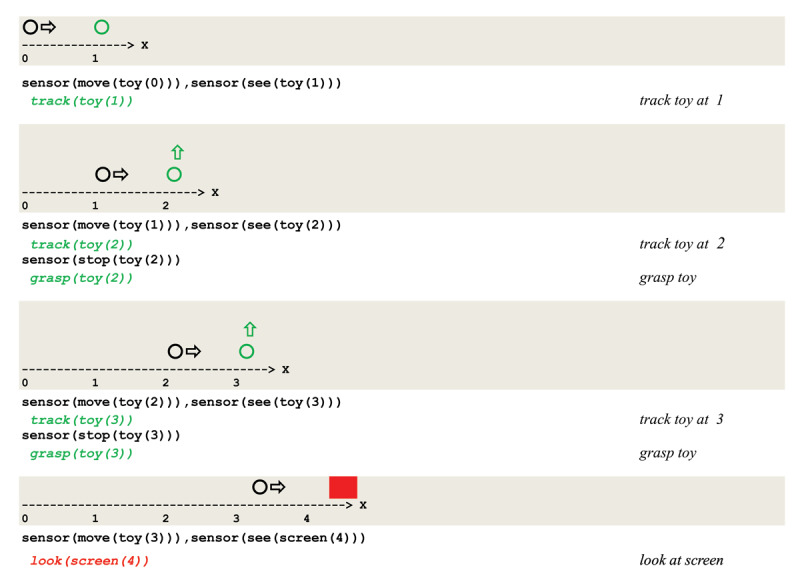
Execution trace of tracking a mobile object.

This is illustrated in Figure 10 containing the execution trace of an actual simulation run.

### 5.4 Modeling visual object tracking and searching (sensory-motor substage 4)

The next substage is marked by a child’s ability to search for an object outside of his visual field e.g., behind a screen. At the beginning, the child does not take into account successive object displacements i.e., for him “*the place where the object was found for the first time remains the place where it will be found*”, leading to the so-called *A not B error*. Piaget proposed a mix of possible explanations for this phenomenon, including a lack of ability to recall the sequence of displacements, to correctly take into account their order, and to separate objects from their context. This has been summarized as resulting from the persistent association binding an object with the infant’s immediate action (Müller et al 2001), or as reflecting the sustained visual attention that accompanies a first reach (Ruffman 2001). In terms of neural processes, this could result from a failure to inhibit a previous response (Munakata 1998; Diamond 2001) i.e., in other terms to bind successive related sensory inputs and actions. After a while, a correct sequential tracking is steadily observed.

**Figure 11:**
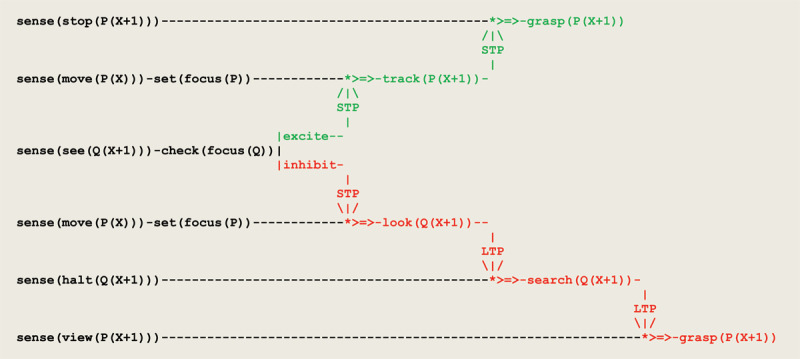
Virtual circuit implementing the visual tracking and searching of a moving object.

A virtual circuit implementing the tracking and searching of a moving object that extends the circuit of Figure 9 is given in Figure 11. This circuit illustrates another example of assimilation and accommodation. The sensation produced by a suspended attention, represented by the sensory input from **sense(halt(Q(X+1)))**, is followed by a new accommodation i.e., a **search** that gets activated by long time potentiation **LTP**. Two different **LTP** models (see Figure 4) reflecting a form of *weak* vs. *strong* form of synaptic plasticity (i.e., a brain maturation that allows at the end for binding successive related sensory inputs and actions) are used here to reproduce in turn an A not B error and a correct sequential tracking. Furthermore, the sensation produced by uncovering an object is assimilated by another sensory input from **sense(view(P(X+1))** that produces a grasping reflex.

In addition to the previous circuit,

- the thread **search(Q(X+1))** gets driven whenever the toy disappears;
- two different models of **LTP** can be used to reproduce an A not B error or a correct sequential tracking;
- signal that object **P** has been uncovered at location **X+1** drives a grasping reflex;
- this grasping reflex is activated by a potentiation from the **search** thread.

**Figure 12:**
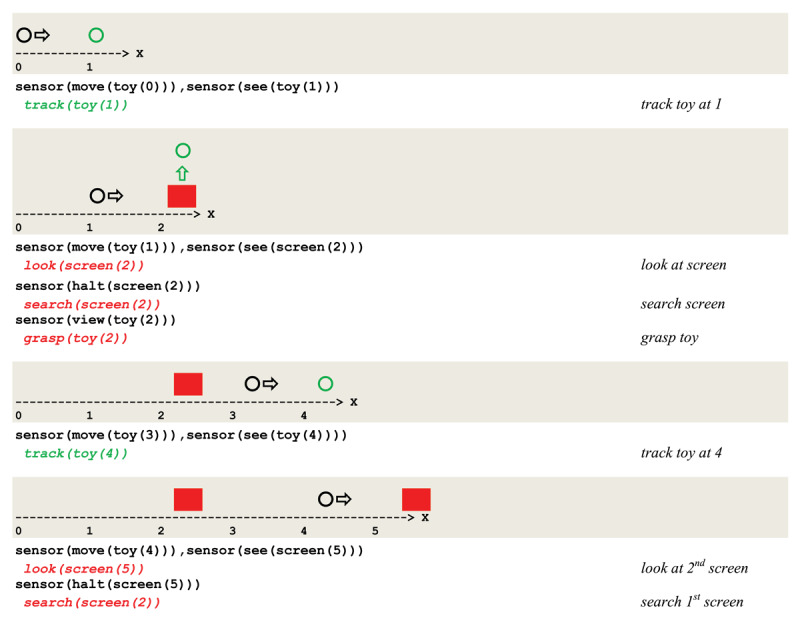
Execution trace of tracking and searching a mobile object with an A not B error.

An execution trace of this circuit implementing a *weak* **LTP** potentiation is given in Figure 12.

**Figure 13:**
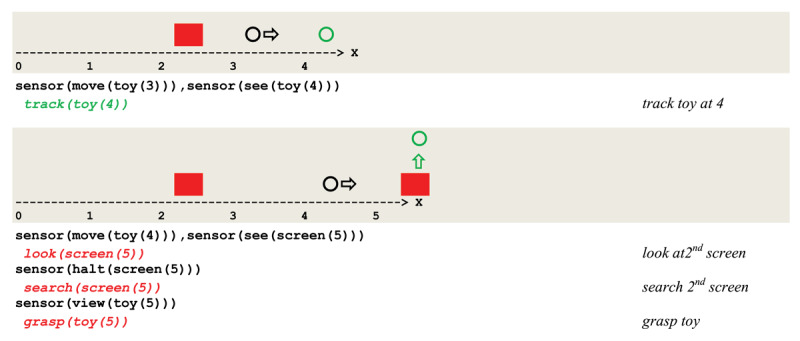
Execution trace of a correct tracking and searching a mobile object.

The unfeasibility of binding a second pair of related inputs at the second screen forced a renewed search at the first screen, thus producing an A not B error. In contrast, the same circuit implemented with a *strong* long time potentiation allows for successive associations of related sensory inputs and actions and produces the execution trace given in Figure 13, which reflects a correct second search.

### 5.5 Modeling a partially invisible displacement (sensory-motor substage 5)

The first acquisition of the next stage is to account for sequential displacements, i.e., to correct the A not B error. As discussed above, this is achieved in our framework by activating the **search** thread through a long time **LTP** potentiation implementing a *strong* from of synaptic plasticity. In order to study the dissociation of objects from their context (e.g., when an object’s position is not directly perceived because of some invisible part along its way), Piaget (1937, p. 75) devised a series of experiments: “*hiding an object not directly under a screen, but in box without a lid; box and object are made to disappear under a screen and the box brought out empty”*. He then observed what he called an “*empirical or practical apprenticeship*” which, he argued, does not yet involve any image or representation of spatial relations.

Our developments follow closely observation 55 from Piaget (pp.75–76). This observation was divided in three phases:

- An object is put in a box while the infant watches; the box is then placed under a screen and turned down to leave the object hidden under the screen without the infant noticing it; the box is finally brought out empty. The infant then searches for the object in the box, eventually looks around, but doesn’t search for the object under the screen
- After a few repetition of this technique followed by the same negative result, the box is left under the screen with the object inside; the infant then immediately looks under the screen and grasps the object (NB. in the original description, the infant finds and grasps the box, *opens it*, and takes the object out of it; these details will be ignored here for the sake of simplicity, especially since the box was not explicitly said to be closed)
- Finally, the experiment protocol of phase I is resumed: this time, the infant first looks for the object in the box and not finding it then searches under the screen (NB this positive result is steadily observed only after a few experiments).

The outcome of phase III led Piaget to conclude that mastering partially invisible displacements (NB which are generally but oddly referred to as “visible displacements”) could not occur through the awareness of some relation or image, but as a result of a “practical schema” acquired through some kind of learning.

**Figure 14:**
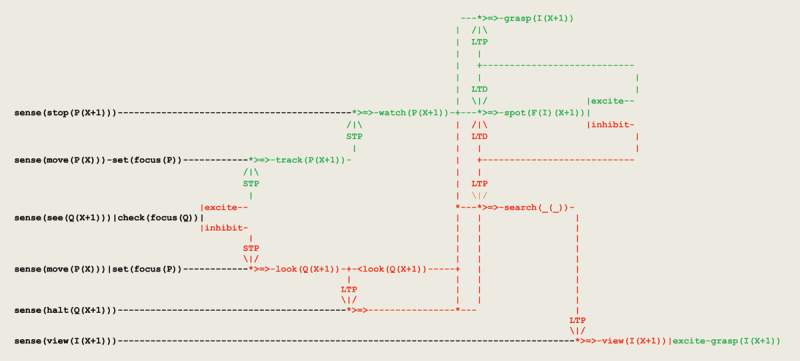
Virtual circuit implementing a partially invisible displacement.

These three stages can be implemented through a further differentiation of the previous schema that gives rise to the schema in Figure 14. In this circuit, the practical learning envisioned by Piaget is implemented as a simple case of *operant conditioning*, which involves a **watch** and a **spot** thread (see Figure 5). This behavior relies on the remembered position where the object equivalently, did disappear, represented in our formalism by a short term memory **<look(Q(X+1))>**.

In addition to the previous circuit,

- the **stop** thread activates a learning circuit where **I** stands for the object contained in box **F**;
- this sub-circuit is imbedded in the overall circuit such that the **search** is now driven by the **halt** thread;
- this search relies on the memorized position **<look(Q(X+1))>** where the object did disappear;
- the object **I** captured by the sensory input **sense(view(I(X+1)))** gets discriminated.

The successive phases of observation 55 have been reproduced in simulation run based on the circuit of Figure 14 (see Figure 16 in the ***Supplementary information*** section).

### 5.6 Modeling invisible displacement (sensory-motor substage 6)

The transition between substages 5 and 6 i.e., when a child starts mastering invisible object displacements, demonstrates a shift from perceptual to representational responses i.e., a capacity that can be invoked in the absence of a perceived reality (McCune 2001). This is summarized as being able to “keep an object in mind” when it is not in sight. This capacity builds up to the sequential tracking of objects that undergo successive invisible displacements. Our developments reproduce here Piaget’s observation 64, translated however in a different involving a covered box instead of a closed hand. This observation is divided in three phases retaining their original numbering.

**Ia.** An object is put in a box and the box is covered by a lid while the infant watches; the box is then placed under a screen and emptied to leave the object hidden under the screen without the infant noticing it; the box is finally brought out empty. The infant searches for the object in the empty box, and then goes on searching for it under the screen
**Ib.** The same experiment is repeated, with the covered box being passed and emptied in a different screen; the infant immediately searches this second screen.
**II.** The experiment protocol of phase I is resumed, but this time the box passes under two successive screens before stopping; the infant looks for the object under the first screen, and not finding it searches the second screen.

**Figure 15:**
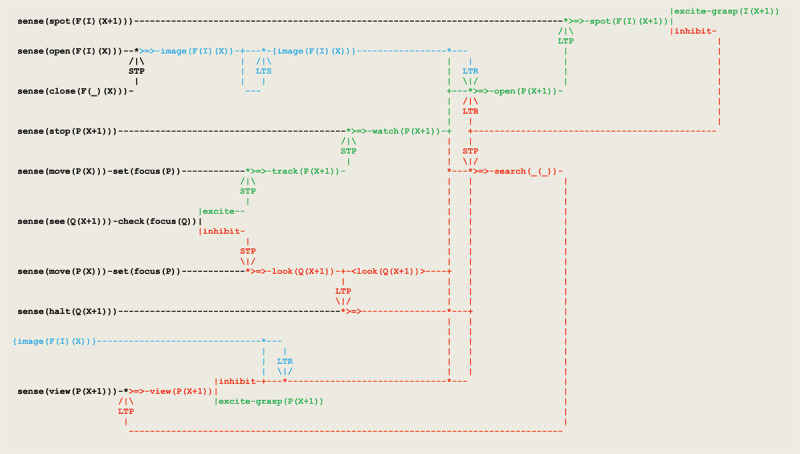
Virtual circuit implementing invisible displacements.

These three phases can be implemented through an ongoing differentiation of the schema in Figure 14 that ends up with the extended circuit in Figure 15.

In addition to the previous circuit,

- sensation gets assimilated by **sense(open(F(I)(X)))** and **sense(close(F(_)(X)))**;
- this sensation gets accommodated in an internal representation **{image(F(I)(X)))}**;
- a retrieval process leads the **watch** thread to activate the opening of the closed box after it stopped;
- a **LTB** process blocks subsequent box openings;
- the **sense(spot(F(I)(X)))** thread leads to either grasp a wanted item or activate a new search;
- a retrieval from **{image(F(I)(X))}** drives the **view** thread to activate a renewed **search**.

At the start, watching the experimenter while he places a toy in a box (or equivalently takes it in his hand palms) and then covers the box (or closes his hands) produces a new sensation involving a *relation* between two objects. This gets assimilated by **sense(open(F(I)(X)))** and **sense(close(F(_)(X)))** threads, where **F** and **I** stand respectively for the box and object, and accommodated by the **image(F(I)(X))** thread that creates an internal representation **{image(F(I)(X))}** implemented via an **LTS** long term storage process. In our formalism (see Figure 6), the internal representation **{P}** of a thread **P** extends the mechanism of long term potentiation and allows for an **LTR(P,Q,R)** retrieval process to be fired by **Q** in order to relate **Q** and **R**, thus defining the basic mechanisms of an associative memory. In the present context, this retrieval process drives the **watch** thread (which stands here for **Q**) to activate the **open** thread (which stands for **R**) and thus trigger the opening of the box after it stops.

After opening the box, **sense(spot(F(I)(X)))** drives **spot** thread to either grasp a desirable item or activate a new search. According to Piaget’s observation, infants who at first do open a box and find it empty do not open it again in subsequent trials. This is achieved here through a long term blocking process **LTB**. Altogether, this new accommodation enlarges the previous operant conditioning learning process by allowing it to be driven by an image evocation.

Finally, in order to take into account successive invisible displacements, an evocation from the **{image(F(I)(X))}** memory drives an **LTR** retrieval process that activates a renewed **search** via the discriminating **view** thread. In order to recall the sequence of displacements and take into account their order, the memory **<look(Q(X+1))>** is implemented as a classical *first-in-first-out* (FIFO) data structure.

This implementation raises the question about possible links between abstract neurological processes and the psychological model they aim to represent. Piaget’s early theoretical developments about mental representations can be found in (Piaget 1936, p.242), where he argues as follows:

*“Hence the accommodation of this stage is more refined than that of the schemata hitherto under study, since the mobile schema applies to relations between external things and no longer only to things in their mere connection with the activity itself”*.

He then goes on asking the question

*“Does this accommodation involve representation?*

to which, after a digression, he proposes to add an additional criterion i.e., that representation must be understood “*to mean the capacity to evoke by a sign or a symbolic image an absent object or an action not yet carried out”*.

According to this argumentation, a criterion for the existence of a mental representation is the capacity of a symbolic image to evoke an action not yet carried out. This capacity is implemented in our virtual neurological framework through the additional functionalities required for tracking invisible displacements recalled above i.e.:

*a) assimilating* a relation between two things through a symbolic image

*b) accommodating* this relation by enabling its retained image to drive an action.

It can be concluded that these additional functionalities characterize mental representations as opposed to mere action schemas, and thus implement a shift from perceptual to representational responses. As postulated by various authors (e.g., Vosgerau 2006; Orlandi, 2020), these representations are in an isomorphic format. In support of this evolution, Ramsay and Campos (1978) demonstrated that infants who had reached substage 6, and thus kept in mind the hidden object, expressed surprise when the toy they uncovered was different.

The successive phases of observation 64 have been reproduced in a simulation run based on the circuit of Figure 15 (see Figure 17 in the ***Supplementary information*** section).

## 5. Summary and conclusion

In his reflection (Poggio 2012, p.7) about the “levels of understanding” framework (Marr & Poggio 1977), Poggio poses the following question: “*did intelligence, as the ability to learn, evolve from associative reflexes and memories with the addition of (neurally simple) primitive operations such as composition of different memories?*”. In order to answer this question, psychological theories should try and relate cognitive concepts to abstract brain structures and processes on which they could be possibly grounded. Failing to do so somehow amounts to putting the cart before the horse.

Piaget’s theory of cognitive development, which is based on schemas that supposedly drive a child’s activity, is detached from any hypothesis about their neurological grounding. Furthermore, this theory is based on his rejection of *association* as a basic mechanism for cognitive development. He introduced instead the collective effect of *assimilation* (i.e., the insertion of new perceptions into schemas) followed by *accommodation* (i.e., the modification and/or extension of schemas), two concepts that are situated at a higher explanatory level than associative memories, and which rely on the preexistence of innate schemas i.e., structures and processes that ultimately have to be grounded at a lower level.

As we have demonstrated in this work, action schemas involved in the sensory-motor stage of this theory can be simulated in a computational framework. These simulations successively allowed for reproducing:

- the grasping of an object in sight (substages 1–2);
- the visual tracking a moving object (substage 3);
- the *A not B* error and the correct retrieval of a hidden object (substage 4);
- the tracking partially invisible object displacements (substage 5);
- the tracking invisible object displacements (substage 6).

The transition to substage 6 represents a shift from perceptual to representational responses. Whereas the implementation of the first substages relies on short/long term potentiation, this last transition requires the additional abstracted neuronal functionality of an associative memory. As defined in our formalism, the **lts/ltr** operations precisely allows for two threads **P** and **Q** attached to separate streams to be associated in order to trigger a recall thread **R**, thus enabling the retained image of a previously perceived relation to drive an action, and thus more generally to implement the basic mechanisms of memory engrams (Queenan et al., 2017; Gallistel, 2021).

This raises the question of which additional basic neural functionalities, if any, would be needed in order for an extended framework to accommodate epistemological concepts, such as beliefs, which presumably involves mental representations in a propositional format (Newen and Vosgerau, 2020). It has been argued that such higher level constructions are unlikely to be based on mechanisms directly related to the lowest level of brain structures and processes (Heyes, 2012). Darwin (1871) himself once wrote that “*Nevertheless the difference in mind between man and the higher animals, great as is it, certainly is one of degree and not of kind*”, thus somehow precluding the existence of fundamental neurological mechanisms that would be unique to humans. When discussing the modalities of mental representations associated with beliefs, Newen and Vosgerau (2020) content themselves with noting that “*the best we can expect is a cluster of neural correlates embedded in one quite contextually varying mechanism or even embedded in a plurality of mechanisms*”. Indeed if, as noted in (Carew 2002, p. 806) and supported by (Baxter et Byrne, 2006) as well by the abstract models presented above, classical and operand conditioning “*have features in common, an exciting principle might emerge: evolution may have come up with a neural ‘associative cassette’ that can be used in either type of conditioning, depending of the neural circuit in which it is embedded*”.

## Data accessibility statement

I confirm that there is no Data Accessibility statement for this work.

## Additional File

The additional file for this article can be found as follows:

10.5334/joc.249.s1 Supplementary information. Execution traces of actual simulation runs.
