# Supplementary material for: Grounding Mental Representations in a Virtual Multi-Level Functional Framework
Source: J Cogn. 2023 Jan 12;6(1):6. doi: 10.5334/joc.249 (PMC9838229; doi:10.5334/joc.249)
Supplement: Supplementary information. — Execution traces of actual simulation runs. [file joc-6-1-249-s1.pdf]

Supplementary information

Execution traces of actual simulation runs.

The execution traces of simulation runs reproducing experiments 55 and 64 from Piaget (1937) are given below.

Experiment 55

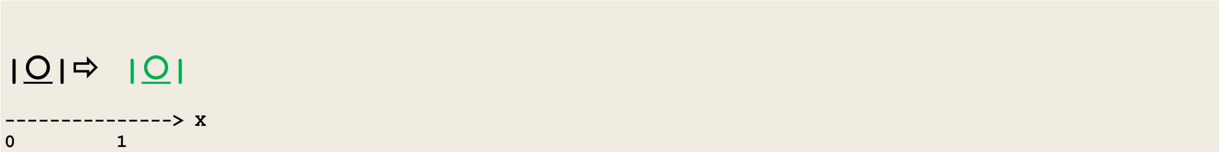

`sensor(move(box(toy)(0))),sensor(see(box(toy)(1)))`	
`track(box(toy)(1))`	*track toy in box at 1*

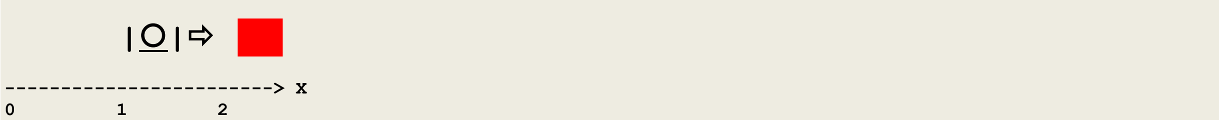

`sensor(move(box(toy)(1))),sensor(see(screen(2)))`	
`look(screen(2))`	*look at screen*

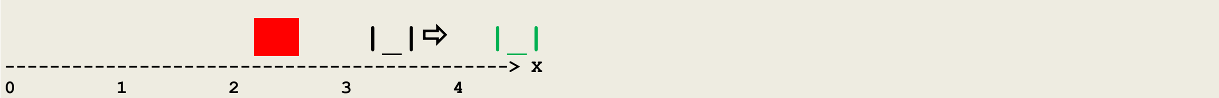

`sensor(move(box([])(3))),sensor(see(box([])(4)))`	
`track(box([])(4))`	*track empty box at 4*
`sensor(stop(box([])(4)))`	
`watch(box([])(4))`	*watch empty box*
`spot(box([])(4))`	*spot empty box*

Fig. 16a. Execution trace of tracking a partially invisible displacement (phase I)

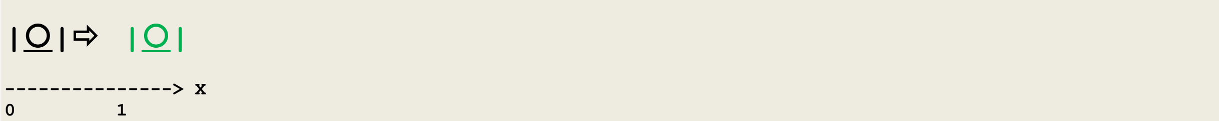

sensor(move(box(toy)(0))),sensor(see(box(toy)(1)))	
track(box(toy)(1))	*track toy in box at 1*

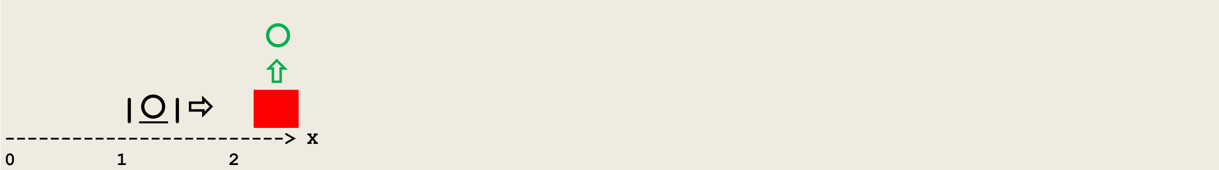

sensor(move(box(toy)(1))),sensor(see(screen(2)))	
look(screen(2))	*look at screen*
sensor(halt(screen(2)))	
search(screen(2))	*search screen*
sensor(view(toy(2)))	
view(toy(2))	*view toy*
grasp(toy(2))	*grasp toy*

Fig. 16b. Execution trace of tracking a partially invisible displacement (phase II)

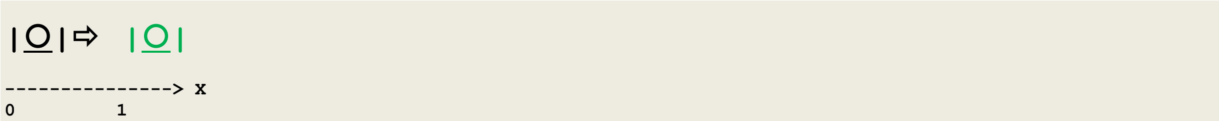

sensor(move(box(toy)(0))),sensor(see(box(toy)(1)))	
track(box(toy)(1))	*track toy in box at 1*

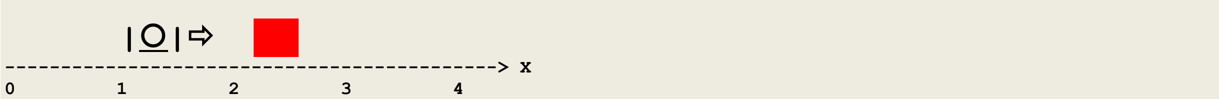

sensor(move(box(toy)(1))),sensor(see(screen(2)))	
look(screen(2))	*look at screen*

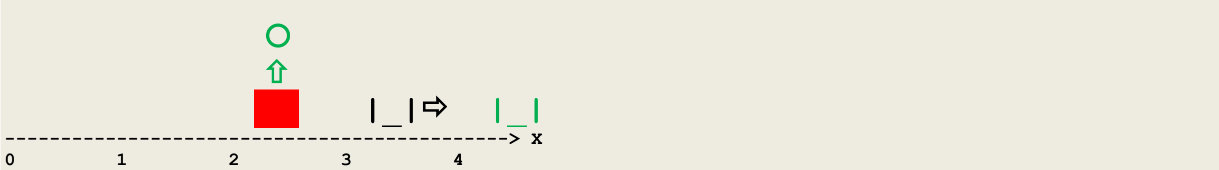

sensor(move(box([])(3))),sensor(see(box([])(4)))	
track(box([])(4))	*track empty box at 4*
sensor(stop(box([])(4)))	
watch(box([])(4))	*watch empty box*
spot(box([])(4))	*spot empty box*
search(screen(2))	*search screen*
sensor(view(toy(2)))	
view(toy(2))	*view toy*
grasp(toy(2))	*grasp toy*

Fig. 16c Execution trace of tracking a partially invisible displacement (phase III)

Experiment 64

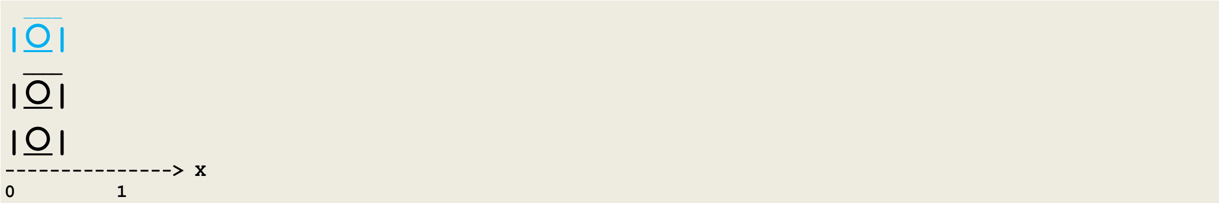

`sensor(open(box(toy)(0))),sensor(close(box(_)(0)))`	
`{image(box(toy)(0))}`	*image of toy in box*

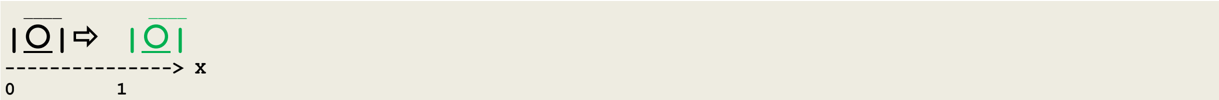

`sensor(move(box(_)(0))),sensor(see(box(_)(1)))`	
`track(box(_)(1))`	*track closed box at 1*

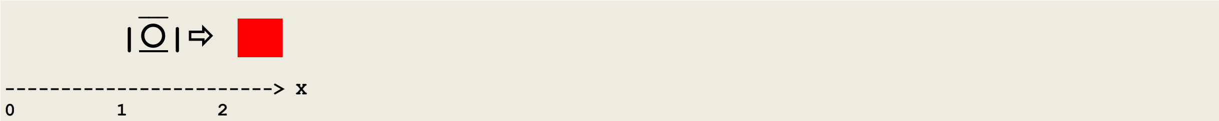

`sensor(move(box(_)(1))),sensor(see(screen(2)))`	
`look(screen(2))`	*look at screen*

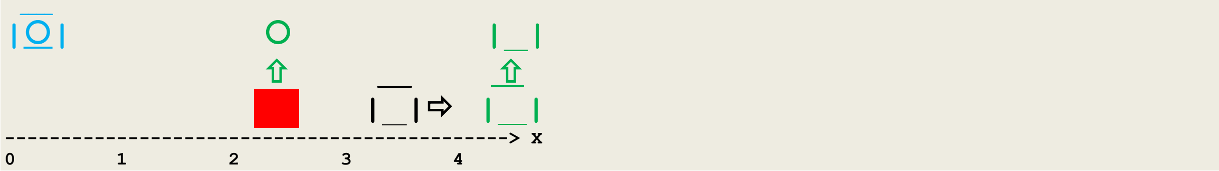

`sensor(move(box(_)(3))),sensor(see(box(_)(4)))`	
`track(box(_)(4))`	*track closed box at 4*
`sensor(stop(box(_)(4)))`	
`watch(box(_)(4))`	*watch closed box*
`{image(box(toy)(0))}`	*evoke mental image*
`open(box(_)(4))`	*open box*
`sensor(spot(box([])(4)))`	
`spot(box([])(4))`	*spot empty box*
`search(screen(2))`	
`sensor(view(toy(2)))`	*search screen*
`view(toy(2))`	*view toy*
`grasp(toy(2))`	*grasp toy*

Fig. 17a. Execution trace of tracking a invisible displacement (phase Ia)

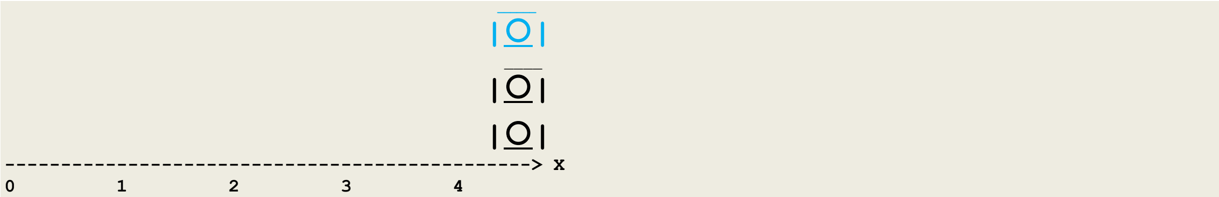


```
sensor(open(box(toy)(4))),sensor(close(box(_)(4)))
 {image(box(toy)(4))}
```
image of toy in box

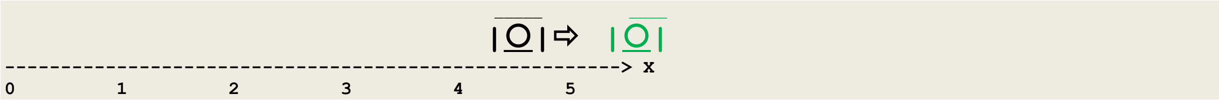


```
sensor(move(box(_)(4))),sensor(see(box(_)(5)))
 track(box(_)(5))
```
track closed box at 5

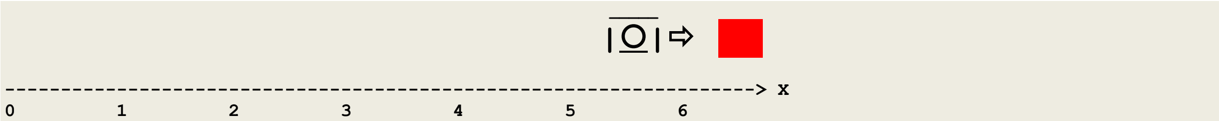


```
sensor(move(box(_)(5))),sensor(see(screen(6)))
 look(screen(6))
```
look at screen

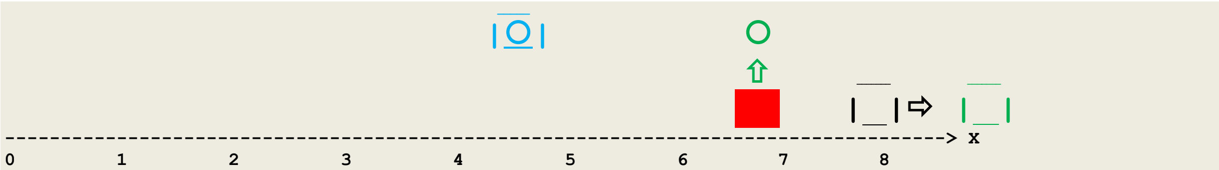


```
sensor(move(box(_)(7))),sensor(see(box(_)(8)))
track(box(_)(8))
sensor(stop(box(_)(8)))
 watch(box(_)(8))
 {image(box(toy)(4))}
 search(screen(6))
sensor(view(toy(6)))
 view(toy(6))
 grasp(toy(6))
```
track closed box at 8

watch closed box
evoke mental image
search screen

view toy
grasp toy

Fig. 17b. Execution trace of tracking of invisible displacement (phase Ib)

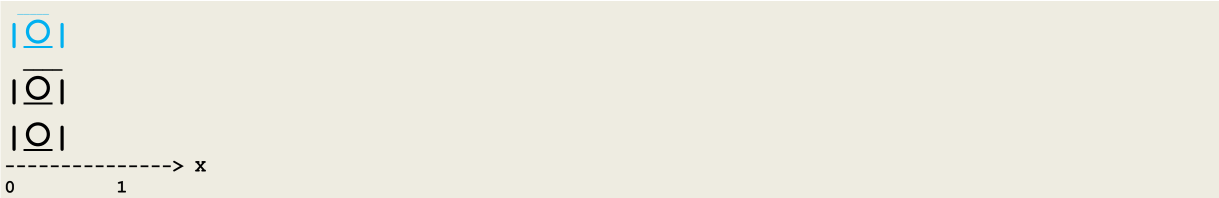

sensor(open(box(toy)(0))),sensor(close(box(_)(0)))
{image(box(toy)(0))} — *image of toy in box*

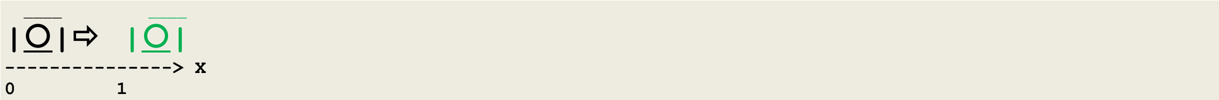

sensor(move(box(_)(0))),sensor(see(box(_)(1)))
track(box(_)(1)) — *track closed box at 1*

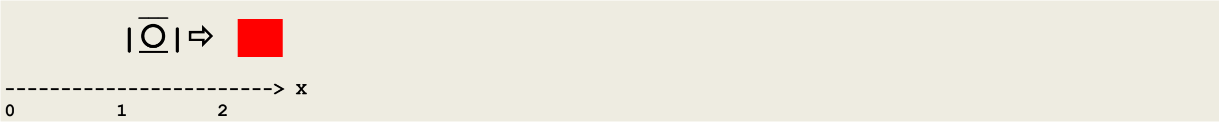

sensor(move(box(_)(1))),sensor(see(screen(2)))
look(screen(2)) — *look at 1st screen*

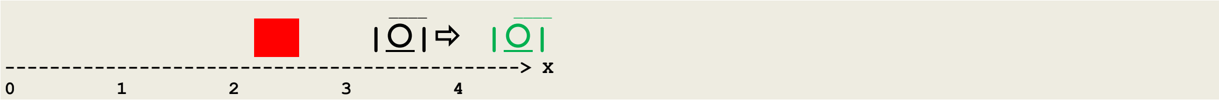

sensor(move(box(_)(3))),sensor(see(box(_)(4)))
track(box(_)(4)) — *track closed box at 4*

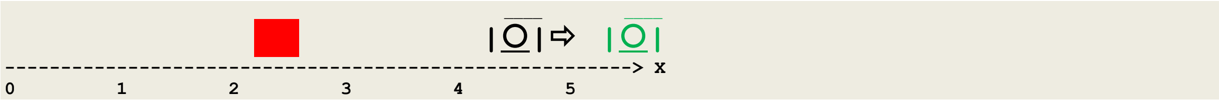

sensor(move(box(_)(4))),sensor(see(box(_)(5)))
track(box(_)(5)) — *track closed box at 5*

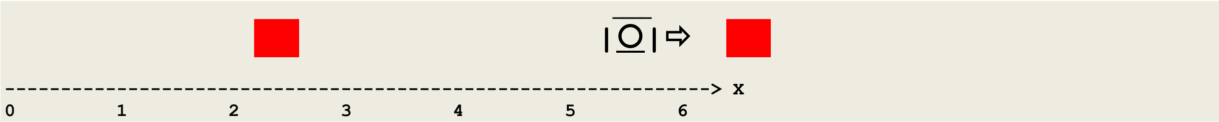

sensor(move(box(_)(5))),sensor(see(screen(6)))
look(screen(6)) — *look at 2nd screen*

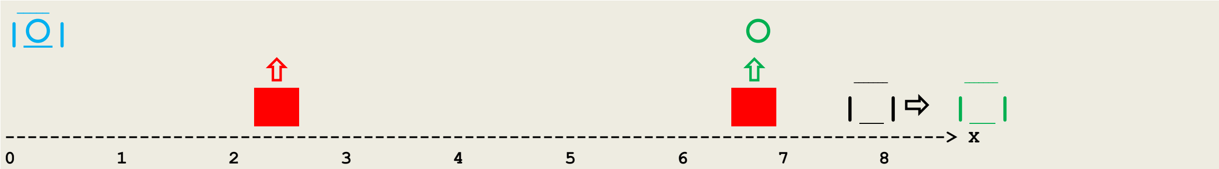

sensor(move(box(_)(7))),sensor(see(box(_)(8)))
track(box(_)(8)) — *track closed box at 8*
sensor(stop(box(_)(8)))
watch(box(_)(8)) — *watch closed box*
{image(box(toy)(0))} — *evoke mental image*
search(screen(2)) — *search 1st screen*
sensor(view([](2)))
view([](2)) — *view void screen*
{image(box(toy)(0))} — *evoke mental image*
search(screen(6)) — *search 2nd screen*
sensor(view(toy(6)))
view(toy(6)) — *view toy*
grasp(toy(6)) — *grasp toy*

Fig. 17c. Execution trace of tracking invisible displacement (phase II)
